# The Dentists' Act, 1921

**Published:** 1923-01

**Authors:** 


### THE DENTISTS' ACT OF 1921 AND ITS RELATION TO THE PUBLIC.

THE Act of last year is an effort to protect the public from the effects of the failure of the Dentists' Act of 1878. The Act of 1878 provided for the efficient training and examination of students setting out to become Dentists. It also sought to preclude the possibility of the untaught and unqualified practising as Dentists. In this last it failed, as was proved by a decision in the House of Lords in 1909, which allowed the untaught and unqualified to evade the spirit of the Act, and thus it failed to protect the public. It had been hoped that this Act would result in the creation of a profession of duly qualified men, but when it was found that the unqualified and inefficient could without cost and expenditure of years in training compete with those who had undergone the special training prescribed by the Act, only a luke warm attention was given to the new profession by the possible student. The professional status of the Dentist, which was to have been made secure by the Act, was so affected by the unprofessional conduct, such as advertising and canvassing, of the uneducated and unregistered, that few could be found to enter the profession, and so what the Act set out to secure for the protection of the public, it failed to obtain, in that it failed to protect the profession. The outcome was an enormous amount of unqualified practice both by companies and individuals all over the country, which discouraged entry into the profession of those the Act sought to encourage.

The position thus created became such a scandal that in 1918 a departmental Committee was appointed to inquire into the whole matter. Its report proved to the hilt the presence of the evil, and as a result the Dentists' Act was passed on July 28, 1921, and it came into force on November 30, 1922.

The new Act seeks to get over the difficulty by a compromise. It provided for the registration of a large body of the unqualified selected, by the application of certain rules, from a much larger body of claimants for registration. This sifting of the applicants for registration as Dentists took place between the dates of the passing of the Act in July, 1921, and its coming into force on November 30, 1922. The work of examining these claims for registration was passed to a Dental Board, by that almost autocratic body, the General Medical Council. This body has hitherto set a standard for examination for the different qualifications obtainable by both Medical and Dental Students. It registered them as Students, and also legally qualified them to practice, and again it disciplined them as to behaviour.

In future the Dental Board, supervised by the General Medical Council, of which it is an off-shoot, will undertake the care of the Dental Register. The qualified of the original register received his qualifications from his College, and was registered in consideration of his agreeing to certain rules governing professional conduct. It is hoped that the concession of registration given to the unqualified by the Act will lead to a cessation of the misconduct found to be prevalent by the departmental inquiry alluded to above, and the Government hope to obtain protection for the public which the inquiry held to be necessary.

From November 30, 1922, no unregistered person can practise dentistry, and by the Act, "the practice of dentistry shall be deemed to include the performance of any such operation, and the giving of any such treatment, advice, or attendance as is usually performed or given by dentists, and any person who performs any operation or gives any treatment, advice or attendance on or to any person as preparatory to, or for the purpose of, or in connection with the fitting, insertion, or fixing of artificial teeth shall be deemed to have practised dentistry within the meaning of the Act."

A person registered under the Act, "(*a*) shall by virtue of being so registered be entitled to take and use the description of dentist or dental practitioner, (*b*) shall not take or use or affix to or use in connection with his premises any title or description reasonably calculated to suggest that he possesses any professional status or qualification other than a professional status or qualification which he in fact possesses and which is indicated by particulars entered in the register in respect to him."

